# Spatio-Temporal Variation of Health Production Efficiency Considering Environmental Pollution in China Based on Modified EBM and Spatial Econometric Model

**DOI:** 10.3389/fpubh.2021.792590

**Published:** 2021-12-31

**Authors:** Fan Liu, Gen Li, Ying Zhou, Yinghui Ma, Tao Wang

**Affiliations:** ^1^School of Business Administration, Zhongnan University of Economics and Law, Wuhan, China; ^2^School of Economics and Management, Jiangsu University of Science and Technology, Zhenjiang, China

**Keywords:** health production efficiency, Spatio-temporal variation, improved EBM model, spatial econometric model, Moran's I

## Abstract

In order to strengthen the construction of China's health industry and improve the health of the people, based on the data of 31 provinces and cities in China from 2009 to 2019, the improved EBM model is used to measure the health production efficiency of each region, and Moran index is used to study the Spatio-temporal variation of health production efficiency of each province. Finally, the spatial econometric model is applied to study the influencing factors of the Spatio-temporal variation of health production efficiency. The results show that generally speaking, the average efficiency of 31 provinces and cities is above 0.7, and the average efficiency of some regions is above 1. From the perspective of time variation, the average efficiency value in the eastern region and the middle region increases from 0.816 to 0.882 and from 0.851 to 0.861, respectively. However, the average efficiency value in the western region and northeast region decreases from 0.861 to 0.83 and from 0.864 to 0.805, respectively. From the perspective of spatial distribution, HH agglomeration and LL agglomeration exist in most regions. By comparing Moran scatter plots in 2009 and 2019, it is found that the quadrants of most regions remain unchanged, and LL agglomeration is the main agglomeration type in local space. There is a significant spatial dependence among different regions. From the perspective of spatial empirical results, *Pgdp, Med*, and *Pd* have a positive effect on health production efficiency. The direct effect and indirect effect of *Pgdp, Med*, and *Gov* all pass the significance test of 1%, indicating that there are spatial spillover effects of the three indicators. Each region should reasonably deal with the spillover effect of surrounding regions, vigorously develop economic activities, carry out cooperation with surrounding regions and apply demonstration effect to accelerate the development of overall health production.

## Introduction

The economy of China is developing rapidly, and the economic level is rising day by day. Under the current high-speed development, we also need to pay attention to the high-quality development of the economy. The rapid economic development has had a great impact on the residents' life, and the modern social civilization has changed the residents' life in many ways. The concept of healthy production has been more mentioned. The report of the 19th National Congress of the Communist Party of China put forward “the implementation of Healthy China Strategy.” Promoting high-quality development of health care is an integral part of the implementation of the Healthy China Strategy and the only way to meet people's growing health needs. According to the data, the national economy in the first quarter of 2021 is generally stable and the development quality is steadily improving. According to the preliminary calculation, the GDP of the first quarter is 24,931 billion yuan, up 18.3% year on year. Meanwhile, the scale of China's health service industry reaches 8 trillion yuan in 2020, accounting for 6.5% of GDP. Market forecast analysis of ChinaIRN has announced that the big health industry will usher in a real outbreak period from 2021 to 2025. In order to respond positively to the Healthy China Strategy, the healthy production level in China on the premise of considering environmental pollution is calculated and the strategies conducive to the development of healthy production are put forward by this paper.

According to the existing research literature on healthy production, scholars mostly use DEA and SFA to measure health production efficiency. For example, Yu Jiali et al. (1) used the DEA-Malmquist model, the decomposable Theil index, and the spatial (traditional) econometric model to study the dynamic evolution of Chinese residents' health production efficiency and its influencing factors from 2002 to 2017. The model was used to calculate the comprehensive efficiency, scale efficiency, and pure technical efficiency of Chinese residents' healthy production. Shi Zhen et al. (2) combined water consumption with wastewater discharge, pollutant concentration in sewage, local medical expenditure, and other factors, and incorporated them into the water resources, energy, and health measurement model, using DEA model to calculate the total efficiency, production efficiency, and health efficiency of 30 provinces from 2014 to 2017. The results show that the key impact indicators were different in each province, and each province should formulate different policies according to its own specific situation, deepen the energy, economic and medical reform in each province, and promote sustainable economic development while improving health efficiency. Shen Shuguang et al. (3) applied SFA to investigate the health production efficiency of various provinces and cities in China from 2010 to 2014 and analyzed the influencing factors of health production efficiency. The study found that since 2010, the health production efficiency in China had improved and regional disparities had narrowed. Based on the data of 30 provinces in China from 2011 to 2017, Shi Zhen et al. (4) adopted the improved undesirable dynamic network SBM model to investigate the economic production stage efficiency and sewage treatment stage efficiency of each province. The results showed that the stage efficiency of sewage treatment was the main factor affecting the overall efficiency. Provinces should therefore follow key indicators to improve efficiency according to local conditions. Ye Li et al. (5) combined the fixed Malmquist-Luenberger (ML) index with the SBM model considering unexpected output, A new slack based measure-Malmquist-Luenberger model was proposed to measure the level of green development in the Pearl River Delta region from 2005 to 2018. The changes of the green development level were analyzed from time and space dimensions. Li Xiangqian et al. (6), Zhang Ning et al. (7), Ma Yuan et al. (8), and Laurie J. Bates et al. (9) all used DEA to study health production efficiency. The above scholars improve and innovate DEA and SFA to make them more consistent with the problems studied, and their conclusions also lay a solid foundation for the research of this paper. In order to study radial ratio and non-radial relaxation improvement simultaneously, the EBM model is used to measure health production efficiency.

At present, there are few studies on the Spatio-temporal variation of health production efficiency, and some scholars have conducted studies on the Spatio-temporal variation of carbon emissions, innovation efficiency, and ecological efficiency. In order to explore the relationship between urban innovation efficiency and high-quality economic development from the perspective of total factors, Yang Weili et al. (10) took Shaanxi province as an example to analyze the static and dynamic characteristics of urban innovation efficiency from 2008 to 2018 and conducted a Spatio-temporal analysis of the coupling relationship between urban innovation efficiency and high-quality economic development: The urban innovation efficiency was effective in most cities, and the growth rate of urban innovation efficiency presented a fluctuating upward trend. The coupling coordination degree between urban innovation efficiency and high-quality economic development increased steadily on the whole. Based on the coupling coordination degree model and factor analysis model, Liu Zhihua et al. (11) took the data of 30 provinces and cities in China from 2001 to 2017 as an example to build a comprehensive evaluation index system for the coupling coordination of carbon emission reduction, economic growth and environmental protection at the provincial level and analyzed the Spatio-temporal variation and driving mechanism of the coupling coordination degree. From the perspective of time, the coupling coordination level of the three systems at the provincial level gradually improved, but it was still in a low state. From the perspective of space, the overall trend was “higher in the Southeast region and lower in the Middle and Western regions,” and the agglomeration was poor. Guo Fuyou et al. (12) comprehensively constructed the evaluation index system of green development in the Yellow River Basin and studied the Spatio-temporal variation characteristics and driving factors of green development in the eco-economic corridor of the Yellow River Basin from 2005 to 2017 by using a variety of measurement methods such as entropy method, spatial autocorrelation analysis, and geographic detector model. The results showed that the green development of the Yellow River Basin mainly came from the external driving effect of large-scale expansion and total growth, and it was inevitable that the endogenous driving effect would be insufficient and unsustainable if the green development of the Yellow River Basin focused on the development speed and ignored the convolution improvement of quality and efficiency. At present, the research on the Spatio-temporal variation of green economy and sustainable development in the academic circle has gradually matured, which provides valuable experience for the research on the Spatio-temporal variation of health production efficiency from the perspective of spatial correlation and spatial heterogeneity in this paper.

According to the current works of literature, most scholars choose to influence factor variables from the aspects of government support, technological level, medical and health care, population density, and so on. He Guanjie et al. (13) adopted the sub-grade index of hospital visitors as a proxy variable of residents' health status and selected regional ecological efficiency and six other control variables as explanatory variables to construct a support vector sensitivity measurement method (SMM-SVM). Li Xiangqian et al. (14) showed that government input capacity and urbanization level had a negative effect on national health production efficiency, and service accessibility had a positive effect on national health production efficiency. Ding Jingmei et al. (15) used DEA to measure the efficiency of the medical and health system in 31 administrative regions of China except for Hong Kong, Macao, and Taiwan and discussed its influencing factors by general linear regression. The results suggest that policymakers should take into account the socio-economic development level and population composition and make targeted changes to the amount of input. Gao Qiuming et al. (16) studied the impact of hospital efficiency on medical service equity based on a proprietary hospital characteristic data set and 630,000 inpatient records from 149 public hospitals in a representative city in China. The results showed that efficiency-oriented health care policies may lead to the loss of social benefits. Wu Haitao et al. (17) found that environmental pollution inhibited the development of comprehensive urbanization, population urbanization, economic urbanization, and urbanization of living conditions in China, but promoted the development of urbanization of the living environment. However, with the increase of residents' health costs, the inhibition effect of environmental pollution on China's comprehensive urbanization, population urbanization, economic urbanization, and housing urbanization was gradually strengthened, while the inhibition effect on housing environment urbanization was weakened. Based on considering the impact of environmental pollution on human health, Zhao Kai et al. (18) extended and constructed the endogenous economic growth model of production, research and development, human capital cultivation, and resource development, and reinterpreted the driving force of China's economy from the perspective of human health by using the public panel model and the space panel model. The study found that China's economic growth relied on physical capital investment and energy consumption. It can be seen from the research conclusions of the above scholars that among the influencing factors, the level of economic development has a positive effect on health production efficiency, and the increase of population density can provide residents with more medical and health services under a moderate financial budget (19). Policy support and health care level can promote the health of the domestic floating population (20). The above research results are numerous, and the selected influencing factors are representative, which is conducive to the construction of the influencing factor index system in this paper. However, the spillover effect is not analyzed and the transmission effect between regions is also worth pondering. This paper will explore the regional transmission mechanism of influencing factors of health production efficiency from the perspective of spatial spillover effect.

After summarizing the above literature, this paper puts forward the following improvements: (1) Environmental pollution is considered in the construction of the input-output index system, and the amount of medical waste produced and the mortality rate are selected as the unexpected output variables. (2) In order to make up for the limitations of the DEA model, this paper decides to use the EBM hybrid model including radial and non-radial distance functions to analyze regional differences in health production efficiency of various provinces and cities. (3) The spatial econometric model is used to analyze the spatial spillover effects of the influencing factors of health production efficiency.

## Research Design

### Measurement Model of Health Production Efficiency in China

In this paper, the super-efficiency EBM model is used to measure the health production efficiency of Chinese residents considering environmental pollution. Existing studies on the measurement of health production efficiency are mainly based on radial or non-radial CCR, BCC, and SBM models in traditional DEA models, but all of them have certain deficiencies: Radial model requires all input-output factors to be reduced or expanded in the same proportion, which is not in line with reality. Although the non-radial model considers the problem of relaxation, the loss rate is the proportion of information between the target input-output value and the actual value (21). In 2010, Tone and Tsutsui put forward the EBM model, which considered both radial ratio and non-radial relaxation improvement quantity, eliminated the coarse and extract the essence of the radial model and non-radial model, kept the radial ratio of the projection value and the original value of the elements and took into account the relaxation variable of the difference of each element. Therefore, in this paper, the EBM model considering the unexpected output will be adopted. Due to the efficiency value of most decision-making units may exceed 1, Andersen et al. (22) established the super-efficiency DEA model to further compare and analyze all decision-making units and realize the calculation of effective decision-making units. In this paper, based on the research of Han Jieping et al. (23), the programming formula of the super-efficiency EBM model which is improved based on non-guidance and considering non-expected output is constructed:


(1) $$\begin{array}{l} \begin{array}{c} \gamma^{*}=\min\frac{\theta -\varepsilon^{-}\overset{m}{\underset{i=1}{\sum }}\frac{w_{i}^{-}s_{i}^{-}}{x_{ik}}}{\varphi +\varepsilon^{+}\left(\overset{s}{\underset{r=1}{\sum }}\frac{w_{r}^{+}s_{r}^{+}}{y_{rk}}+\overset{q}{\underset{p=1}{\sum }}\frac{w_{p}^{u-}s_{p}^{u-}}{u_{pk}}\right)}\\ i=1,2,...,m;r=1,2,...,s;p=1,2,...,q\\ s.t\overset{n}{\underset{j=1,j\neq k}{\sum }}\lambda_{j}x_{ij}+s_{i}^{-}\leq \theta x_{ik}\\ \overset{n}{\underset{j=1,j\neq k}{\sum }}\lambda_{j}y_{ij}-s_{r}^{+}\geq \varphi y_{rk}\\ \overset{n}{\underset{j=1,j\neq k}{\sum }}\lambda_{j}u_{pj}+s_{p}^{+}\leq \varphi u_{pk}\\ \lambda_{j}\geq 0,s_{i}^{-},s_{r}^{+},s_{p}^{+}\geq 0 \end{array} \end{array}$$

Where, γ^*^ represents the health production efficiency of the province. *k* represents the number of decision units, and *x*_*ik*_, *y*_*rk*_, *u*_*pk*_ represents the input, expected output, and unexpected output of the *k* decision unit, respectively. $s_{i}^{-},s_{r}^{+},s_{p}^{u-}$ represent the relaxation variables of input, expected output, and unexpected output, respectively; $w_{i}^{-},w_{r}^{+},w_{p}^{u-}$ represent the weight of each input indicator, expected output and non-expected output indicator; θ and φ are radial components of γ^*^; ε is a key parameter with a value range of [0~1], indicating the importance of the non-radial part. When ε = 0, the ultra-efficient EBM model is equivalent to the radial model; when θ = ε = 1, the ultra-efficient EBM model is equivalent to the non-radial SBM model.

### Spatial Correlation Analysis Method of Health Production Efficiency in China

In order to explore the spatial correlation of health production efficiency in China, this paper applies the spatial analysis method (24) to study its regional impact. Firstly, the global Moran's I (25) is selected to reveal the spatial distribution pattern of health production efficiency (26). The calculation formula is as follows:


(2) $$\begin{array}{l} I=\frac{n\overset{n}{\underset{i=1}{\sum }}\overset{n}{\underset{j=1}{\sum }}W_{ij}|\gamma_{{\;}_{i}}^{*}-\bar{\gamma^{*}}||\gamma_{{\;}_{i}}^{*}-\bar{\gamma^{*}}|}{\overset{n}{\underset{i=1}{\sum }}\overset{n}{\underset{j=1}{\sum }}W_{ij}\overset{n}{\underset{i=1}{\sum }}|\gamma_{{\;}_{j}}^{*}-\bar{\gamma^{*}}{|}^{2}} \end{array}$$

In Formula (2), $\gamma_{{\;}_{i}}^{*},\gamma_{{\;}_{j}}^{*}$ is the efficiency value of each spatial unit, $\bar{\gamma^{*}}$ is the average efficiency value of each spatial unit, and *W*_*ij*_ is the spatial weight matrix of each spatial unit. Finally, the spatial correlation between various regions can be judged according to the positivity and negativity of Moran's I.

The global Moran's I can measure the global spatial correlation of observed variables. Considering the instability of local variables, the *Getis-Ord* $G_{i}^{*}$ index is introduced to analyze the spatial aggregation phenomenon between local variables. The calculation formula is as follows:


(3) $$\begin{array}{l} G_{i}^{*}=\overset{n}{\underset{j=1}{\sum }}\left(W_{ij}\gamma_{{\;}_{j}}^{*}\right)/\overset{n}{\underset{j=1}{\sum }}\gamma_{{\;}_{j}}^{*} \end{array}$$

According to the value of the *Getis-Ord* $G_{i}^{*}$ index, the aggregation of each local space is judged.

### Spatial Econometric Model of Health Production Efficiency in China

Currently, the spatial econometric models are commonly used in academic circles to study health production efficiency, including two modes, one is the spatial panel lag model (SLM) and the other is spatial panel error model (SEM) (27).

The spatial lag model mainly discusses whether a certain region has spillover and diffusion effects on its surrounding regions. The following expression of the SLM model is established in this paper according to the influencing factors:


(4) $$\begin{array}{l} \gamma_{{\;}_{it}}^{*}=\rho Wy+X\theta_{it}+\varepsilon_{it} \end{array}$$

Where, $\gamma_{{\;}_{it}}^{*}$ represents the health production efficiency of the *i* province in the year *t*, and ρ is the spatial regression estimation coefficient, which reflects the spatial dependence between the observed values of samples, namely, the observed values of adjacent regions. *W* is the *n*×*n*-order spatial weight matrix, which generally selects the proximity matrix or distance matrix. *Wy* is the spatial lag explanatory variable, representing the observed value of the dependent variable in the surrounding area, reflecting the effect of spatial distance on each space unit. ε is the random error term vector, and *X* is the *n*×*k*-order explanatory variable matrix.

The spatial error model is different from the spatial lag model. The spatial dependence in the spatial error model mainly exists in the error term. The spatial error model is mainly used to reflect the different relative positions between regions. The mathematical expression is:


(5) $$\begin{array}{l} \begin{array}{c} \gamma_{{\;}_{it}}^{*}=X\theta_{it}+\mu_{it}\\ \mu_{it}=\lambda W_{z}+\varepsilon_{it} \end{array} \end{array}$$

Where $\gamma_{{\;}_{it}}^{*}$ represents the health production efficiency of the *i* province in the year *t*, *X* is the exogenous explanatory variable matrix of *n*×*k*, *W* is the spatial weight matrix of *n*×*n*, ε is the random error vector, μ is the random error vector of normal distribution, and λ is the spatial error coefficient of the dependent variable vector.

## Variable Design

### Input-Output Variables

In reference to the relevant works of literature on healthy production, this article selects the total health expenses, per capita expenditure of medical insurance, per capita medical capital stock, and health technical personnel as the input variables, through the analysis of health input indicators, health input index system covers the health, labor, and capital in the field of production elements, the total health expenses and per capita medical capital stock covers the medical equipment investment, the cost such as the construction of medical institutions, so used to measure the health capital investment, health technical personnel to reflect the connotation of human capital investment, per capita expenditure of medical insurance reflect medical spending, it has also been identified as one of the indicators of health investment.

At present, health outcomes are mostly measured using vital statistics. However, there are many gaps in current data on life expectancy that do not reflect continuous levels of healthy production. Perinatal survival rate and mortality rate are highly available indicators and reflect healthy levels of production in the region, the perinatal survival rate as expected output variables. Medical waste production and mortality rate are selected as the unexpected output variables. The relevant variables are explained as follows.

(1) Input variables. ① Total health expenses. Total health cost includes the government, society, and individuals' investment in health and medical services, which is used to describe the health situation of a region. This paper selects total health costs to represent the medical capital investment of each region. ② Per capita expenditure of medical insurance. According to the expenditure scope and expenditure standard stipulated by the national policy, the medical insurance treatment expenses are paid from the social overall planning fund to the employees and retirees participating in the basic medical insurance, and the medical expenses are paid from the personal account fund to the employees and retirees participating in the basic medical insurance, as well as other expenses. Per capita medical insurance is used in this paper. ③ Per capita medical capital stock. In order to measure the input of medical and health materials in each region, this paper selects the index of medical capital stock, which is measured by the number of beds in medical and health institutions per thousand population. ④ Health technical personnel. In this paper, the index of the number of health technical personnel needs to use the relative quantity index, and the index of the number of health technical personnel per thousand population is used. The index can reflect the investment of human capital in health organizations and measure the level of technology in each region.

(2) Expected output variables. In many previous studies, average life expectancy is selected as the output index, but due to the lack of data, the perinatal survival rate is used as the expected output index in this paper. The perinatal mortality rate is often expressed in permillage. Between the 28th week of gestation and the 7th day after birth (or birth weight above 1,000 g), the perinatal mortality rate is the ratio of stillbirths, dead-birth and neonatal deaths caused by fatal diseases or maternal diseases affecting the fetus to survival number of newborns (2). After obtaining the perinatal mortality, the perinatal survival rate (1- perinatal mortality rate) can be converted.

(3) Unexpected output variables. ① Medical waste production. This paper considers the impact of environmental pollution on healthy production. In order to reflect environmental pollution factors in health efficiency, this paper selects medical waste production. In consideration of the availability of data, this paper chooses hazardous waste production data as a substitute. ② Mortality rate. The output variables of health production are mainly related variables such as mortality. In this paper, the perinatal survival rate is selected above, and the population mortality variable is selected to measure the unexpected output.

### Influencing Factor Variables

Based on previous studies, variables of economic development level, the service level of medical institutions, government support system, and population density degree are often selected as influencing factors. Besides, this paper chooses medical non-marketization degree as an explanatory variable by referring to Shen Shuguang et al. (3).

(1) Economic development level

The economic development level of each province is most directly reflected by the GDP index. In this paper, the per capita GDP index is selected. Ochalek Jessica et al. used per capita GDP as an empirical control variable (28). Generally speaking, areas with high economic development levels will have higher social production efficiency, higher social construction level, and higher living standard of people. Therefore, areas with higher levels of health production efficiency will also have higher levels of health production efficiency. It is predicted that per capita GDP will have a positive effect on health production efficiency.

Urbanization level represents the degree of agglomeration of urban residents, which can reflect the urbanization process and the economic development level of each province. It is an important indicator of regional economic development degree. It is also a condition to measure the level of regional social development. It is assumed that the level of urbanization will have a positive effect on health production efficiency.

(2) Service level of medical institutions

The service level of medical institutions determines the length of hospitalization and recovery time of residents, so this paper adopts the average length of hospitalization to measure the service level of medical institutions. Liu Weilin et al. used an average length of hospitalization to represent health resource output utilization (29). The average length of stay in a hospital refers to the average length of stay of each patient in a certain period, which can reflect the management ability and medical technology level of medical service institutions. The larger the average length of stay, the lower the service level of the medical institution, and the smaller the average length of stay, the higher the service level of the medical institution. At the same time, it can save the medical resources of each institution and improve the healthy production level of residents. Therefore, the average length of stay is assumed to have a negative effect on health production efficiency.

(3) Medical non-marketization

Since there are too many public hospitals in the medical market, the market competition is relatively calm, which will reduce the reform and innovation motivation of each hospital. This paper chooses the ratio of public hospitals to private hospitals as a quantitative index of the degree of medical non-marketization. Private hospitals can play a role in causing benign competition in the market, which is conducive to intensifying competition in the medical market and causing public hospitals to carry out reform and innovation, so as to promote the development of the medical market and improve the medical level of each region. This paper assumes that the degree of medical non-marketization has a negative effect on health production efficiency.

(4) Government support system

Government policies in each region determine the development direction of the region. Government support for healthy production can be measured by government financial health expenditure, and the relative quantity is selected as the index. In this paper, the proportion of government financial health expenditure in GDP is chosen as the expression. Cheng Zhaohui et al. used government financial health expenditure as environment variables for empirical research (30). Government financial expenditure specifically refers to the financial allocation of health undertakings by local governments, which is generally used for public health service funds and free medical funds. Government financial health expenditure has an important influence on regional public health construction levels. The more government financial health expenditure, the higher the level of medical and health facilities construction is. It is assumed that government expenditure on health has a positive effect on health production efficiency.

(5) Population density

Population density refers to the population per unit of land, which is one of the important indices for regional population distribution. It influences the output level of regional healthy production. On one hand, high population density would have a burden on the local medical and health institutions, but at the same time, the higher the population density, regional production efficiency will be higher. Maniaci Antonino et al. (31) and Chandra Bharatendu et al. (32) showed that long-term use of N95 protective masks in the work of medical staff would reduce the work level of medical staff, and larger public hospitals would make more complete rules in this regard. Combining previous research on the index, it assumes that population density has a positive effect on health production efficiency. To sum up, the selected variables are summarized, as shown in Table 1.

**Table 1:** Variable description.

**Variable**	**Symbol**	**Definition**
Expected output	*y*	Perinatal survival rate (‰)
Capital investment	*K_*e*_*	Total expenditure on health (million yuan, Ln, based on 2009)
	*K_*i*_*	Per capita medical insurance (ten thousand yuan, based on 2009)
	*K_*b*_*	Number of beds in medical and Health institutions per thousand population (sheets)
Human input	*I*	Number of health technicians per thousand population (persons, Ln)
Unexpected output	*B_*w*_*	Medical waste production amount (ten thousand tons)
	*B_*m*_*	Mortality rate (%)
Per capita GDP	*Pgdp*	GDP/Population by region (ten thousand Yuan/person, based on 2009)
Urbanization level	*Urb*	Urbanization level (%)
Service level of medical institutions	*Med*	Average length of stay in hospital (days)
Medical non-marketization	*Mnm*	Number of public/Private hospital establishments (%)
Government support system	*Gov*	Fiscal health expenditure /GDP (%)
Population density	*Pd*	Area population/Area (People/km^2^)

### Sample Selection and Data Sources

In this paper, the data of 31 provinces and cities in China from 2009 to 2019 are selected for the analysis of health production efficiency. Due to the lack of some data in Tibet and Hong Kong, Macao, and Taiwan, the study is not included. The data of all indicators come from the China Statistical Yearbook, China Health Statistical Yearbook, China Urban Statistical Yearbook, and relevant statistical yearbook data of all provinces and cities. For some missing data, interpolation and weighted average are used to complete the data. Descriptive statistics of relevant variables are shown in Table 2. Table 2 shows that there are large inter-provincial differences in the amount of medical waste produced and population density.

**Table 2:** Descriptive statistics of variables.

**Variable**	**Minimum**	**Maximum**	**Standard deviation**	**Mean**
Perinatal survival rate (‰)	0.760	0.982	0.034	0.936
Total expenditure on health (million yuan, Ln, based on 2009)	3.519	8.662	0.903	6.789
Per capita medical insurance (ten thousand yuan, based on 2009)	0.044	0.598	0.101	0.225
Number of beds in medical and Health institutions per 1,000 population (sheets)	2.390	7.550	1.192	4.871
Number of health technicians per thousand population (persons, Ln)	2.370	15.460	2.027	5.794
Medical waste production amount (ten thousand tons)	0.050	1046.04	173.690	140.849
Mortality rate (%)	0.042	0.076	0.008	0.060
per capita GDP (ten thousand Yuan/person, based on 2009)	1.1062	16.1642	2.561	4.832
Urbanization level (%)	0.223	0.896	0.136	0.555
Service level of medical institutions (days)	8.1	16.2	2.401	9.923
Degree of medical non-marketization (%)	0.1976	32.000	2.7254	1.4347
Government support system (%)	0.0074	0.0753	0.0119	0.0213
Population density(People/km^2^)	2.4104	3853.9683	683.6284	450.4962

## Spatio-Temporal Variation of Health Production Efficiency in China

### Health Production Efficiency in China

In this paper, MAXDEA (Professional edition) software (Beijing Rewomaidi Software Co., LTD, Beijing, China) was used to calculate the health production efficiency of 31 provinces in China. The results are shown in Table 3. According to the National Bureau of Statistics' economic regional division of China, 31 provinces are divided into four regions: Eastern region, Middle region, Western region, and Northeastern region. Table 3 describes the health production efficiency of 31 provinces and cities from 2009 to 2019. The health production efficiency of 31 provinces and cities each year can be seen intuitively.

**Table 3:** Health production efficiency in China 2009-2019.

**CRS**	**2009**	**2010**	**2011**	**2012**	**2013**	**2014**	**2015**	**2016**	**2017**	**2018**	**2019**	**Max**	**Min**	**Mean**	**Region**
BeiJing	1.017	1.027	1.026	1.012	1.009	1.008	1.000	1.012	1.005	0.752	0.728	1.027	0.728	0.963	Eastern
TianJin	0.696	0.707	0.695	0.758	0.750	0.787	1.004	1.003	1.014	1.010	1.022	1.022	0.695	0.859	Eastern
HeBei	0.744	0.763	0.779	0.846	0.831	0.899	1.000	0.889	0.849	0.835	0.838	1.000	0.744	0.843	Eastern
GuangDong	1.019	1.030	1.034	1.024	1.027	1.027	1.037	1.037	1.036	1.032	1.031	1.037	1.019	1.030	Eastern
HaiNan	1.074	1.114	1.107	1.092	1.066	1.081	1.080	1.099	1.097	1.093	1.090	1.114	1.066	1.090	Eastern
ShangHai	0.655	0.816	0.747	0.787	0.758	0.820	0.872	1.004	0.904	0.830	0.765	1.004	0.655	0.814	Eastern
JiangSu	0.687	0.708	0.708	0.743	0.730	0.780	0.789	0.778	0.773	0.804	0.845	0.845	0.687	0.758	Eastern
ZheJiang	0.748	0.768	0.781	0.803	0.790	0.804	0.812	0.807	0.804	0.804	0.797	0.812	0.748	0.793	Eastern
FuJian	0.822	1.012	1.006	0.943	0.807	0.833	0.903	0.876	0.893	0.893	0.978	1.012	0.807	0.906	Eastern
ShanDong	0.706	0.717	0.705	0.710	0.734	0.724	0.738	0.727	0.715	0.725	0.723	0.738	0.705	0.720	Eastern
ShanXi	0.735	0.846	0.780	0.789	0.801	0.788	0.887	0.868	0.879	0.892	0.819	0.892	0.735	0.826	Middle
AnHui	1.002	1.008	1.008	0.975	1.008	1.004	1.002	1.002	1.003	1.008	1.013	1.013	0.975	1.003	Middle
JiangXi	1.073	1.032	1.044	1.028	1.024	1.025	1.020	1.023	1.011	1.008	1.004	1.073	1.004	1.027	Middle
HeNan	0.808	0.853	0.846	0.845	0.876	0.832	0.862	0.878	1.003	1.007	0.807	1.007	0.807	0.874	Middle
HuBei	0.761	0.824	0.830	0.805	0.804	0.758	0.798	0.760	0.753	0.763	0.783	0.830	0.753	0.785	Middle
HuNan	0.730	0.751	0.747	0.758	0.761	0.755	0.768	0.752	0.757	0.765	0.743	0.768	0.730	0.753	Middle
GuangXi	0.866	1.001	0.891	0.798	0.804	0.784	0.810	0.809	0.806	0.820	0.810	1.001	0.784	0.836	Western
ChongQing	1.005	0.822	0.838	0.825	0.872	0.812	0.830	0.790	0.780	0.760	0.751	1.005	0.751	0.826	Western
SiChuan	0.745	0.740	0.741	0.741	0.815	0.740	0.776	0.754	0.761	0.766	0.763	0.815	0.740	0.758	Western
GuiZhou	1.028	1.029	1.025	1.007	1.010	0.865	0.875	0.838	0.788	0.771	0.761	1.029	0.761	0.909	Western
YunNan	0.758	0.758	0.748	1.002	0.770	0.777	0.784	0.768	0.773	0.785	0.764	1.002	0.748	0.790	Western
XiZang	1.132	1.120	1.143	1.161	1.185	1.189	1.193	1.193	1.190	1.170	1.160	1.193	1.120	1.167	Western
ShaanXi	0.752	0.767	0.786	0.791	0.779	0.765	0.773	0.762	0.757	0.748	0.761	0.791	0.748	0.767	Western
GanSu	0.775	0.786	0.857	0.857	0.877	0.838	0.888	0.855	0.845	0.827	0.787	0.888	0.775	0.836	Western
QingHai	0.821	1.002	0.797	1.001	0.752	0.772	0.782	0.793	0.777	0.780	0.785	1.002	0.752	0.824	Western
NingXia	1.021	1.007	1.016	1.022	1.028	1.027	1.023	1.016	1.012	0.806	0.821	1.028	0.806	0.982	Western
XinJiang	0.697	0.676	0.731	0.741	0.713	0.722	0.749	0.812	0.833	0.860	1.001	1.001	0.676	0.776	Western
NeiMengGu	0.742	0.785	0.790	0.785	0.771	0.770	0.812	0.802	0.789	0.771	0.799	0.812	0.742	0.783	Western
LiaoNing	0.782	0.693	0.715	0.735	0.742	0.752	0.780	0.753	0.755	0.744	0.762	0.782	0.693	0.747	Northeastern
JiLin	1.007	0.749	0.808	0.892	0.959	0.794	0.910	0.838	0.815	0.796	0.798	1.007	0.749	0.852	Northeastern
HeiLongJiang	0.802	1.008	0.801	0.786	0.771	0.778	0.832	0.825	0.816	0.851	0.855	1.008	0.771	0.830	Northeastern

(1) From the Eastern region, the average efficiency of Hainan is the highest at 1.090. The efficiency value of Hainan from 2009 to 2019 is >1, and the comprehensive efficiency value of Guangdong province is also >1 each year, slightly lower than Hainan province. Hainan takes the tertiary industry as the main target of development and implements high-quality and high-standard green development on every project. Therefore, only in this way can the health production efficiency of Hainan be maintained above 1 and the quality of healthy development can be maintained. Hainan and Shandong belong to the eastern region, but there is a big gap in healthy production efficiency, because the annual hazardous waste discharge in Hainan is much lower than that in Shandong, so the efficiency value in Hainan is higher than that in Shandong.

Among other provinces and cities, Jiangsu province's health production efficiency has been on the rise, and Jiangsu province has achieved initial results in the development of healthy output. On the contrary, the health production efficiency of Beijing decreased from 1.005 to 0.752 in 2018. In terms of efficiency decomposition, the scale efficiency of Beijing is 0.748. Therefore, Beijing should pay attention to the rational allocation of medical resources and strengthen the management level of medical and health institutions. From 2009 to 2019, the value of health production efficiency in Shanghai also increases from 0.655 to 0.765. As an important member of the Yangtze River Delta Economic Zone, Shanghai vigorously promotes the policy of healthy development. In 2019, Shanghai Health Promotion Committee issued the “Healthy Shanghai Action (2019-2030).” However, as the health service industry in Shanghai is in the initial stage of development, there is still a huge space for the development of health production in Shanghai.

(2) In the Middle region, the average efficiency values of Anhui and Jiangxi are both >1, indicating a good trend of healthy development. The health production efficiency values of Shanxi and Henan remain near 0.8, with little regional difference. In the Middle region, the fluctuation of health production efficiency of all provinces and cities is not large, and the regional development is relatively stable. The level of economic development in middle China is relatively balanced. The efficiency value of Jiangxi province is the highest and that of Hunan province is the lowest, which is not only related to the medical level of the two provinces but also related to the difference of waste discharge between Jiangxi province and Hunan province. At the same time, the population density of Hunan province is too large, resulting in a huge burden on local medical treatment and a lower efficiency level among the middle region.

(3) The development level of the Western region is relatively low in all regions. The implementation of the “Western Development” policy has driven the economic recovery of the Western region. As the development of the Western region focuses on the primary and secondary industries, the economic recovery has also brought negative impacts on the environment and residents' health. The health production efficiencies of Guangxi, Chongqing, Guizhou, Qinghai, Ningxia are dropping. The elevation and bad natural environment of Tibet and Xinjiang are not conducive to the development of the second industry. At the same time, due to the special natural scenery and tourism service industry, health production efficiency is rising slowly.

(4) In the Northeast region, with the support of the central party committee, the old industrial base has been established since 2004. Health production efficiency values of Liaoning, Jilin, and Heilongjiang in 2019 do not reach 0.9. It stands for the low level of healthy development. The Northeast region needs to draw lessons from foreign areas of heavy industry and explore a characteristic road of healthy development. The average efficiency of Liaoning in Northeast China is the lowest and that of Jilin is the highest. This is related to the level of medical technology in Jilin province, which has more medical technicians than the other two provinces. The excessive discharge of medical waste and heavy environmental pollution in Liaoning led to the decline of efficiency value.

### Time Variation of Health Production Efficiency

#### Regional Analysis of Health Production Efficiency

After exploring the health production efficiency values of 31 provinces from 2009 to 2019, the efficiency values will be divided into four regions, namely the Eastern region, the Middle region, the Western region, and the Northeast region, to explore the inter-regional differences in the development of healthy production.

**Figure 1:**
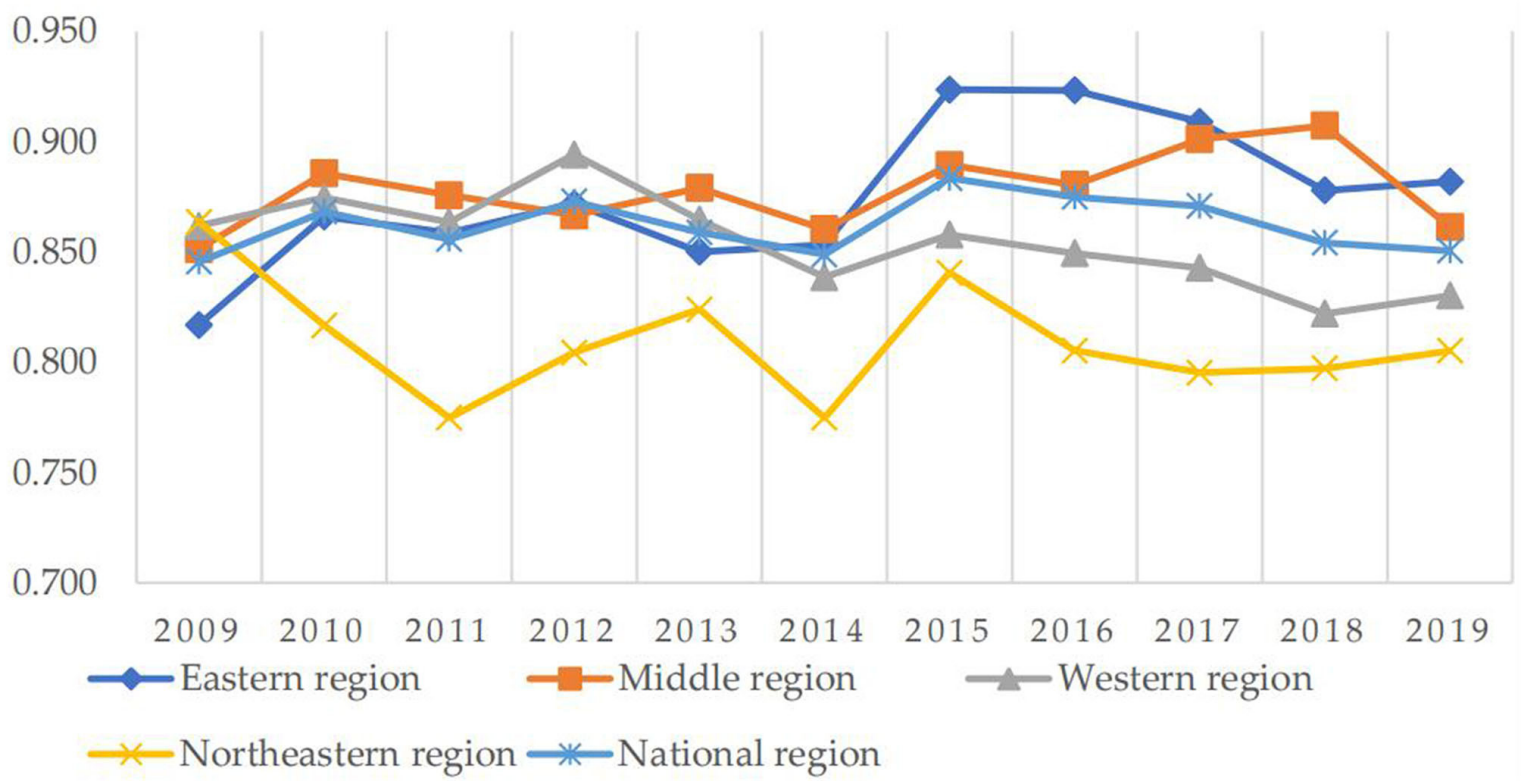
Health production efficiencies of National and four regions from 2009 to 2019.

As shown in Figure 1, the Eastern region has the highest efficiency value in 2019, while the Northeastern region has the lowest efficiency value. The health production efficiency in the Eastern region fluctuates from 0.816 to 0.882. There is a slight downward trend in health production efficiency in both the Western and Northeast regions, and the health production efficiency in the Western region decreases from 0.861 to 0.83. In the Northeast region, it drops from 0.864 to 0.805. The Middle region shows a slow growth trend from 2009 to 2018 but suddenly decreases in 2019. However, there is a slight increase from 0.851 to 0.861 in general. There has been a slow decline in the Western region. The efficiency value in the Northeast region fluctuates greatly. The ups and downs of the efficiency values in the four regions are different, but the dispersion degree of the health production efficiency in each region is not large, and there is little difference in the health production efficiency values in each region.

#### Decomposition Analysis of Health Production Efficiency

The comprehensive efficiency of healthy production is decomposed to explore the role of pure technical efficiency and scale efficiency in the process of healthy production.

**Figure 2:**
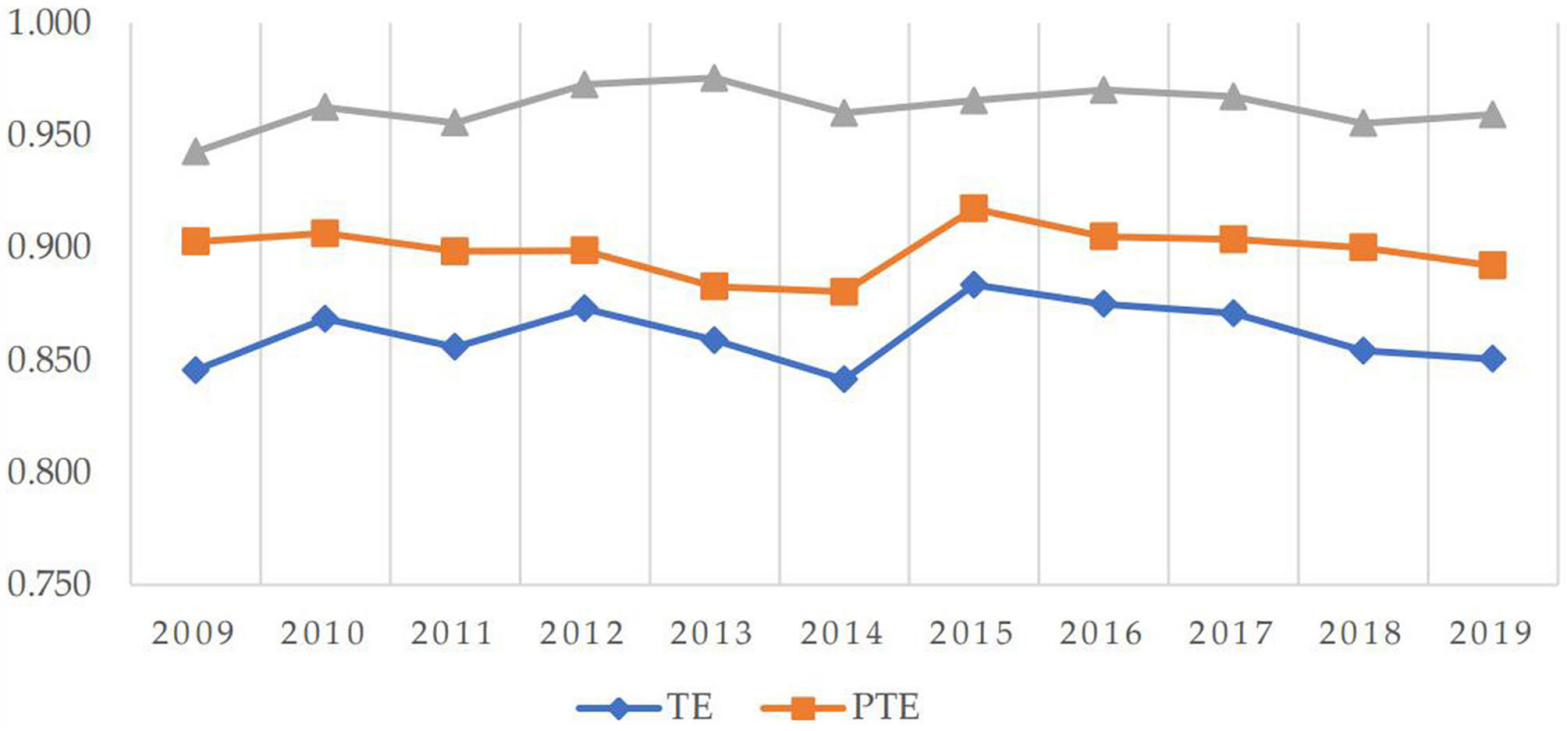
Breakdown of health production efficiency from 2009 to 2019.

As shown in Figure 2, the fluctuation range of the overall efficiency at the national level is relatively small, and there is a similarity in the fluctuation trend of TE and PTE, indicating that the health production efficiency of provinces and cities is greatly affected by technical factors. Before 2009, China's economy was hit by the financial crisis caused by the US subprime mortgage risk, but at the same time, the 2008 Beijing Olympic Games was successfully held, the economy began to recover. China's health production efficiency began to show positive growth. Since 2015, China's health production efficiency had shown a trend of slow decline. In 2015, the haze had increasingly appeared, which had a huge impact on the respiratory health of Chinese residents. With an increase in respiratory diseases, it resulted in a decrease in health production efficiency. Since 2016, the CPC Central Committee and the State Council had issued the outline of the “Healthy China 2030” plan, calling on the whole society to strengthen their sense of responsibility and mission. It encouraged every effort to promote the building of a healthy China. Subsequently, the decline rate of China's health production efficiency began to slow down, and the health production efficiency would definitely return to the upward trend after the reform in the future. From the decomposition results, SE and TE had the same fluctuation trend from 2009 to 2014, indicating that the allocation and utilization of medical resources before 2014 led to the fluctuation of TE, and after 2014, PTE had a major impact on the fluctuation of TE. The future reform should focus on the development of technical level and the improvement of medical resource management level.

### Spatial Variation of Health Production Efficiency

**Figure 3:**
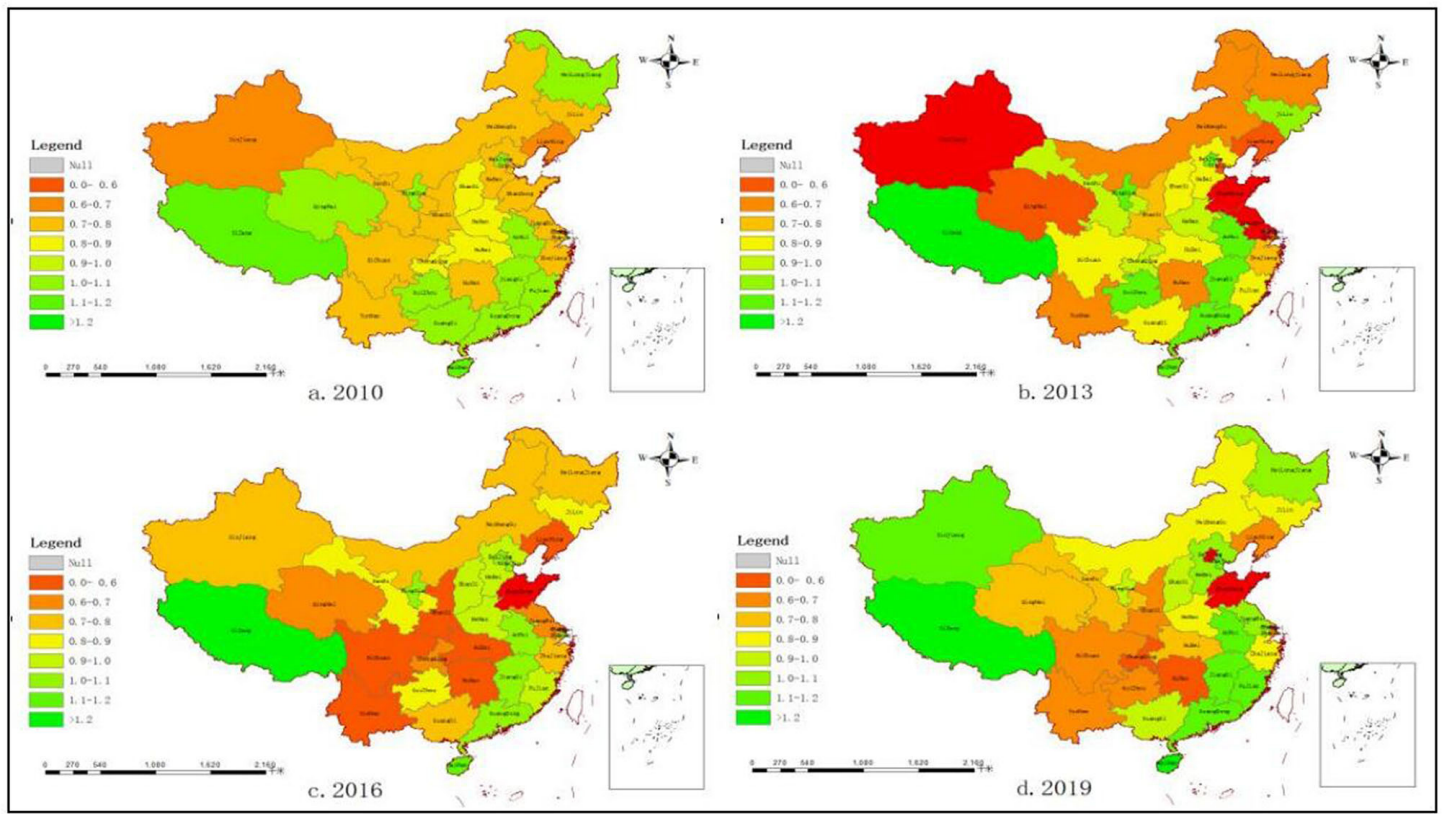
Distribution of health production efficiencies in 31 provinces and cities in China. **(A)** Distribution of health production efficiencies in 2010. **(B)** Distribution of health production efficiencies in 2013. **(C)** Distribution of health production efficiencies in 2016. **(D)** Distribution of health production efficiencies in 2019.

In order to conveniently analyze the Spatio-temporal evolution of health production efficiency values in each region, the efficiency values are divided into eight grades, which is shown in Figure 3. (0, 0.8] is low efficiency, (0.8, 1] is medium efficiency and higher than 1 is high efficiency. Meanwhile, in order to evenly reflect the spatial distribution of efficiency values in each time period, 4 years of 2010, 2013, 2016, and 2019 are selected to draw the map of health production efficiency of 31 Provinces and cities. It can be seen from the map above: (1) Health production efficiencies in Tianjin, Hebei, Shanxi, Shanghai, and Xinjiang are on the rise; Health production efficiencies in Beijing, Jilin, Chongqing, and Ningxia are on the decline. (2) The efficiency value in the Western region has been significantly improved. Tourism in Xinjiang and other regions has developed rapidly and residents' living standards have improved. The distribution of efficiency values in the Middle region shows LL agglomeration. The distribution chart in 2019 shows that most of the Middle region is at a low level. In order to improve the economic level, Anhui, Jiangxi, Hunan, and other regions vigorously develop modern equipment manufacturing and high-tech industries, which have an impact on the environment and lead to the decline of efficiency values. The efficiency value in the Northeast region rises first and then falls. The establishment of the industrial base in the Northeast region improves people's salary level and produces a large amount of waste pollution, which leads to the fluctuation of efficiency value. There is a slight decrease in efficiency in the Eastern region, which is related to the development trend of the Eastern region. While developing the service industry, the maturity of the primary and secondary industries has a double effect on efficiency. The map above visually reflects the spatial change process of health production efficiency in different regions. (3) The distribution of health production efficiency in China changes from low in the Western region, medium in the Middle region, high in the Eastern region, and medium in the Northeast region to high in the Western region, medium in the Middle region, high in the Eastern region, and high in the Northeast region. From the perspective of spatial distribution, there are more efficient regions in China, especially in the Western and Northeast regions. In recent years, the government's policy for the Northeast region has achieved initial results, and the healthy production difference among regions is gradually narrowing.

### Spatial Pattern Texture Features

This paper uses Geoda software (Dr. Luc Anselin and his team, Chicago, IL, USA) to calculate the global Moran index of China's health production efficiency from 2009 to 2019, which is shown in Table 4. The Moran index values are all above 0.1 at the significant level of 10%, indicating that there is a positive spatial autocorrelation. This paper is suitable to use a spatial econometric model to empirically analyze the influencing factors.

**Table 4:** Global Moran index of health production efficiency from 2009 to 2019.

**Year**	**Moran index**	***Z***	***P*-value**
2009	0.152	1.370	0.037
2010	0.106	1.169	0.041
2011	0.091	1.460	0.035
2012	0.108	1.630	0.029
2013	0.161	1.007	0.016
2014	0.147	1.099	0.049
2015	0.119	1.096	0.045
2016	0.145	1.591	0.025
2017	0.117	1.153	0.013
2018	0.183	1.702	0.062
2019	0.186	1.878	0.040

**Figure 4:**
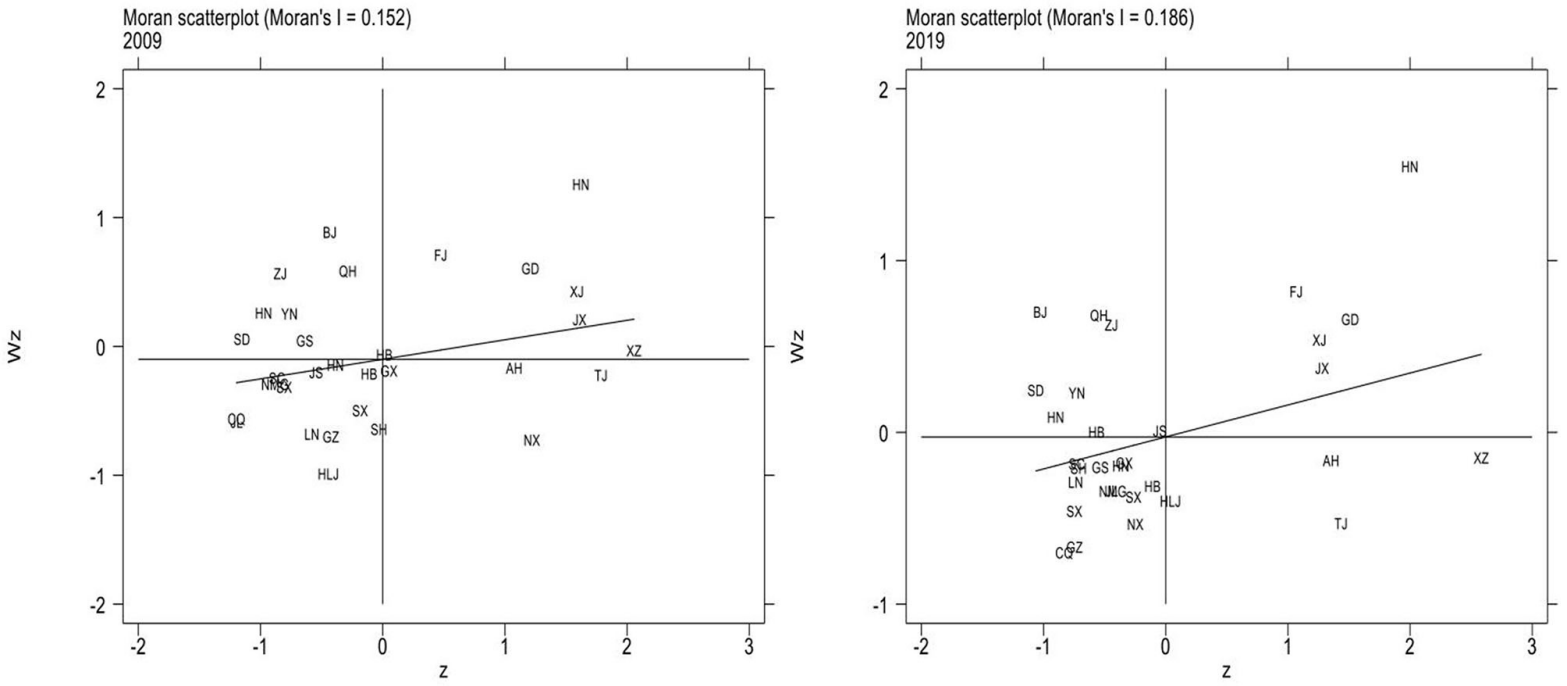
Local Moran scatter plots in 2009 and 2019.

Figure 4 shows local Moran scatter plots of China's health production efficiency in 2009 and 2019. The first quadrant (HH) is the high-level region surrounded by the high-level regions. The second quadrant (LH) is the low-level region surrounded by the high-level regions. The third quadrant (LL) is the low-level region surrounded by low-level regions. Quadrant 4 (HL) is the high-level region surrounded by low-level regions. For brevity, the names of the provinces in Figure 4 are abbreviated. The abbreviations of provinces should be in accordance with the provisions of the Announcement on the Adjustment of China Internet Domain Name System issued by the Ministry of Information Industry.

In the 2009 Moran scatter chart, Hunan, Guangdong, and Xinjiang are high cluster regions, while Shanxi, Liaoning, and Heilongjiang are low cluster regions. According to the 2019 Moran scatter chart, most regions are clustered in the first and third quadrants. Hainan, Fujian, and Jiangxi are high cluster regions in 2019, while Chongqing, Ningxia, and Shanxi are low cluster regions. It can be seen that the quadrants of most regions have not changed. Ningxia has changed from HL to LL, and Tibet from HH to HL. In general, LL agglomeration is the main agglomeration type in local space. Therefore, the spatial distribution of health production efficiency in China is correlated. There is a significant spatial dependence among different regions and the effect between regions is worth further study.

## Influencing Factors of Spatio-Temporal Variation of Health Production Efficiency in China

### Applicability Analysis of the Model

Before conducting a spatial empirical study on data, a unit root test should be carried out on data to measure the stability of data and ensure that no false regression will occur. Since T < N in this paper belongs to the short panel, the HT test method is used (26). In this paper, the stationarity test of six influencing factor variables is carried out by using Stata software (StataCorp, College Station, TX, USA), and the results are shown in Table 5. It can be seen that the test results of the six influencing factor variables reject the original hypothesis and they are significant at the level of 1%, which means that the six influencing factor variables have passed the stationarity test.

**Table 5:** Panel unit root inspection results.

**Variables**	**Statistic**	***Z***	***P*-value**
*Pgdp*	0.8762	−4.6248	0.0000
*Urb*	0.4892	−19.0797	0.0000
*Med*	−0.0078	−37.6424	0.0000
*Mnmt*	0.6321	−13.7406	0.0000
*Gov*	0.4001	−22.4070	0.0000
*Pd*	0.5160	−18.0766	0.0000

According to the Moran index analysis results above, the regions have spatial correlations. Then the spatial panel model is selected, which needs to carry out an LM test on the data. Stata/mp16.0 space measurement software (StataCorp, College Station, TX, USA) was used to complete the following space measurement operations. According to Table 6, LM error and LM lag are significant at the level of 1%. Therefore, the OLS regression is rejected and the spatial econometric model is selected, the value of the spatial error model is greater than that of the spatial lag model. Therefore, the spatial error model is more in line with the empirical analysis of health production efficiency in this paper.

**Table 6:** Test values of LM test statistics.

**Test statistics**	**LM-error**	**LM-lag**	**Robust LM-error**	**Robust LM-lag**
*p*-value	123.585***	119.061***	8.216**	3.693*
	0.000	0.000	0.004	0.055

*Means significant at the 10% level.

**Means significant at the 5% level.

***Means significant at the 1% level.

Then it is necessary to choose to use a random effect model or fixed-effect model. It is determined according to the results of the Hausman test. The results of the Hausman test are 45.9 and significant at the level of 1%. Therefore, the original hypothesis is rejected and the fixed effect model is selected.

According to the above regression results, we can know that the data in this paper have both spatial error effect and spatial lag effect. Therefore, the spatial Durbin model (SDM) is preliminarily selected for empirical analysis. At the same time, it is necessary to test the robustness of the model by running the LR test and Wald test to observe whether the SDM model will degenerate into SEM and SAR. The results are shown in Table 7. The LR test results are significant at the level of 1%, and SDM will not degenerate into SEM and SAM. The Wald test results also show that the SDM model is the most consistent with the empirical model of health production efficiency.

**Table 7:** LR test and Wald test results.

**Model**	**SEM_a**	**SAR_a**
LR	83.41***	91.47***
	0.000	0.000
Wald	49.97***	38.85***
	0.000	0.000

***Represents significant at 1% level.

Then the SDM model is selected for regression, and the time fixed-effect model, individual fixed-effect model, and double fixed effect model are used for regression, respectively. Finally, the R coefficient values of the three regression models are compared. The test results show that the R coefficients of the time fixed-effect model, individual fixed-effect model, and double fixed effect model are 0.2230, 0.1105, and 0.1188, respectively, so the time fixed effect model is selected for regression.

### Regression Analysis of Time Fixed Effect SDM

After the regression model is selected, the data are empirically analyzed by using Stata16.1 software (StataCorp, College Station, TX, USA). The regression results of the SDM are shown in Table 8, and the regression coefficients and significance levels of six influencing factors are obtained.

**Table 8:** Regression results of SDM.

**Variables**	**Main**	**Wx**	**Spatial**	**Variance**
*Pgdp*	0.021***	0.046***		
	(0.00)	(0.00)		
*Urb*	−0.694***	−0.605**		
	(0.00)	(0.05)		
*Med*	−0.028***	−0.037**		
	(0.00)	(0.01)		
*Mnm*	−0.001**	−0.006*		
	(0.05)	(0.08)		
*Gov*	3.339***	7.787***		
	(0.00)	(0.00)		
*Pd*	0.890***	−0.718*		
	(0.00)	(0.06)		
rho			0.074	
			(0.32)	
sigma^2^_*e*				0.003***
				(0.00)
R-squared	0.111	0.111	0.111	0.111

*Represents significant at 10% level.

**Represents significant at 5% level.

***Represents significant at 1% level.

The test result of per capita GDP is significant at the level of 1%, and the coefficient value is 0.021, indicating that per capita GDP has a positive effect on regional health production efficiency. Per capita GDP can measure the level of economic construction in a region. Similar to the conclusion of other scholars, the higher the Per capita GDP, the higher the health production efficiency of the region is. Per capita GDP not only reflects the living standard and consumption capacity of residents but also reflects the economic operation of a region from the macro level. Under a good socio-economic management system, environmental pollution is contained, medical facilities are improved, and people's health is improved, which leads to an increase in health production efficiency value.

The regression coefficient of urbanization level is −0.694, which is significant at a 1% level. The result shows that the higher the urbanization level is, the lower the health production efficiency is, which is contrary to the expected result. The urbanization level reflects the progress of urbanization in a region and stands for the advanced degree of local social organization and management level. It is an important indicator of the process of urban development. The improvement of urbanization level can provide residents with better urbanization services, a perfect medical system, and a mature social supervision system. It is helpful to improve people's lives and health production efficiency. Yet, on the other hand, considering its own special national condition, the improvement of urbanization level also means that the dramatic increase in urban population will make enormous pressure on the health service and community building. The government should deal with the increasing pressure on the development of population and find the right speed of urbanization development to let people really enjoy the benefits of urbanization.

The service level of medical institutions is expressed by the average length of stay, and the result shows that the coefficient of service level of medical institutions is −0.028, which is significant at the level of 1%, meaning that the lower the average length of stay, the higher the health production efficiency level is. It is the same as the expected result. The average length of stay in a hospital not only represents the technical level of the medical and health institutions but also highlights the comprehensive management ability of the medical institutions in the region. The shorter the length of stay, the less the consumption of medical resources is. Reducing the average length of stay in a hospital can not only minimize the cost of medical resources but also save the cost of medical treatment for patients. Medical institutions should be strongly supported to improve service levels in order to increase health production efficiency.

The test result shows that the regression coefficient of the degree of medical non-marketization is −0.001 at the significant level of 5%. The degree of medical non-marketization would have a negative impact on the health production efficiency, which is the same as the expected result. The higher the degree of medical non-marketization, the fewer private hospitals in the medical market are. Too many public hospitals and insufficient market competition are not conducive to the reform and innovation of the medical market, thus reducing the regional health production efficiency. According to the analysis results, the medical marketization degree should be properly improved and the competitiveness of regional medical service institutions should be enhanced.

The regression coefficient of the government support system is 3.339, which is significant at the level of 1%. It is the same as the predicted result. The proportion of government financial health expenditure in GDP can reflect the resources used for health and medical service construction in a certain period of time. The higher the proportion of government financial health expenditure in GDP is, the higher the local government attaches importance to medical and health construction, and the health production efficiency of local residents will increase accordingly.

Population density has dual influences on economic development in each region. If it is too high, it can cause residents living costs to increase, such problems as shortage of resource allocation and commuter crowd. While if population density is too low, it will cause a shortage of labor supply, a declining birth rate, and lower production efficiency. According to the test results of this paper, the regression coefficient of population density is 0.89 at the significant level of 1%, indicating that the population density of China's 31 provinces is in a state of equilibrium, and the uniform distribution of population plays a role in promoting health production efficiency, so it is necessary to balance the population growth rate and economic development rate in the future development.

### Spatial Spillover Effects of Influencing Factors of Health Production Efficiency

In order to further study the spatial spillover effects of the six influencing factors, effect decomposition of the SDM is carried out, and it is found that the direction of the coefficient of total utility is the same as the direction of the spatial Durbin empirical results, as shown in Table 9.

**Table 9:** Effect decomposition results of SDM.

**Variable**	**Direct effect**	**Indirect effect**	**Total effect**
*Pgdp*	−0.0148***	0.0255***	0.0107***
*Urb*	−0.7887***	0.1863	−0.6024***
*Med*	−0.0289***	−0.0715***	−0.1004***
*Mnm*	−0.0010*	−0.0016	−0.0026***
*Gov*	6.9213***	−3.2741***	3.6472***
*Pd*	0.0020	0.0057	0.0077***

*Represents significant at 10% level.

***Represents significant at 1% level.

Population density (*Pd*) does not pass the significance test of direct effect, *Urb, Mnm*, and *Pd* do not pass the significance test of indirect effect. However, *Pgdp, Med*, and *Gov* all pass the significance test of 1%, indicating that the three indicators all have spatial spillover effects. The direct effect of *Pgdp* is negative, and the indirect effect is positive, indicating that the higher the level of *Pgdp* in a certain region has a promotion effect on the health production efficiency of the surrounding regions. The indirect effect of *Med* is negative, that is the higher service level of the medical institutions will cause the loss of health production efficiency in surrounding regions. The higher regional medical service organization level will attract surrounding residents to see a doctor, resulting in medical institutions in surrounding regions is difficult to improve the service level. As a result, it has an inhibiting effect on the health production efficiency of surrounding regions. The indirect effect of *Gov* is negative, indicating that the higher the local government support system is, the lower the health production efficiency of surrounding regions. The direct effect of *Gov* is 6.9213, indicating that government support has a great promoting effect on local health production efficiency, and there is a demonstration effect on surrounding regions.

## Conclusions and Recommendations

Based on the EBM model, Moran index, and SDM, this paper measures and studies the Spatio-temporal variation of health production efficiency in China. The results show that:(1) In general, the average efficiency of 31 provinces and cities is above 0.7, and the average efficiency of some regions is above 1, such as Guangdong, Hainan, Anhui, and Jiangxi. From the results of the Eastern region, there is no clear causal relationship between the value of health production efficiency and the amount of health investment. (2) The spatial correlation of health production efficiency is analyzed. The results show that there are “high” and “low” clustering phenomena of health production efficiency in most regions, and there is a spatial correlation between regions. (3) The data of influencing factors of health production efficiency in 31 provinces of China pass the panel unit root test, indicating the smoothness of the data. From the perspective of spatial empirical results, *Pgdp, Gov*, and *Pd* have a positive effect on health production efficiency. The direct effect and indirect effect of *Pgdp, Med*, and *Gov* all pass the significance test of 1% and the three indicators have spatial spillover effects. At the same time, *Pgdp* can promote the health production efficiency of surrounding regions.

Based on the above research results, this paper puts forward the following countermeasures and suggestions to improve the health production efficiency of provinces and cities in China:

First, the government should strengthen the urbanization of provinces and cities to provide better modern services for residents. Economic and social development is bound to bring urbanization, and in the process of promoting urbanization, it is bound to bring the problem of over-density of the urban population. In order to explore the development path suitable for China's urbanization layout and form, all provinces and cities should actively respond to the implementation of new urbanization policy, and promote the coordinated development of large, small, and medium-sized cities.

Second, the government should promote medical marketization and increase healthy competition in the medical market. From the point of the current status of the medical market, public hospitals occupy most of the medical resources in the market. Public hospitals also have far more credibility in the health care market than private hospitals. It not only can lead to narrow roads in the development of private hospitals but also lead to too much pressure on the public hospital market. The problem of seeing a doctor is difficult to solve. A partial marketization can increase the competitiveness of the medical market, promote reform and innovation in public hospitals and improve the development environment for private hospitals.

Third, the government's financial investment structure in health should be optimized. The decomposition result of the health expenditure effect of government finance shows that government support can promote regional health production efficiency. There is no doubt that public health expenditure has a positive impact on people's health, but more consideration should be given to the coverage of rural areas, and the medical condition gap between rural and urban should be narrowed to achieve comprehensive health coverage for people.

Fourth, in order to improve China's health production efficiency, reduce regional differences, improve China's overall health production efficiency level, the government should also increase government financial investment in health, expand the scale of the health industry, improve the green level of various industries, reduce environmental pollution and improve the health of residents in each region. Each region should make reasonable use of the spillover effect of surrounding regions, vigorously develop economic activities, carry out cooperation with surrounding regions and use the demonstration effect to accelerate the development of overall health production efficiency.

## Data Availability Statement

Publicly available datasets were analyzed in this study. This data can be found here: China Statistical Yearbook, China Health Statistical Yearbook, China Urban Statistical Yearbook and relevant statistical yearbook data of all provinces and cities. For some missing data, interpolation and weighted average are used to complete the data.

## Author Contributions

FL: conceptualization, methodology, and software. GL: data curation, visualization, and investigation. YZ: software, validation, and writing-original draft preparation. YM and TW: reviewing and editing. All authors contributed to the article and approved the submitted version.

## Funding

This paper was funded by the National Natural Science Foundation of China (No. 71503106 and No. 71803197), Jiangsu Social Science Fund (19EYB016), Jiangsu College Philosophy Social Science Outstanding Innovation Team Construction Project, Ministry of Education Humanities and Social Sciences Foundation (No. 18YJC630094), Fundamental Research Funds for the Central Universities (No. 31511910801).

## Conflict of Interest

The authors declare that the research was conducted in the absence of any commercial or financial relationships that could be construed as a potential conflict of interest.

## Publisher's Note

All claims expressed in this article are solely those of the authors and do not necessarily represent those of their affiliated organizations, or those of the publisher, the editors and the reviewers. Any product that may be evaluated in this article, or claim that may be made by its manufacturer, is not guaranteed or endorsed by the publisher.
